# T73. NOVEL VISUAL TRAINING FOR COGNITIVE REMEDIATION IN SCHIZOPHRENIA

**DOI:** 10.1093/schbul/sby016.349

**Published:** 2018-04-01

**Authors:** Toral Surti, Dayshalis Ofray, Bruce Wexler

**Affiliations:** 1 Yale University School of Medicine, VA Connecticut Healthcare System; 2 Yale University School of Medicine

## Abstract

**Background:**

Though cognitive remediation has shown modest benefits for schizophrenia, relatively few training programs for have targeted the visual processing deficits common in the illness. Residual visual processing deficits following cognitive remediation may explain why cognitive remediation has had limited effects on visual learning and memory compared to auditory learning and memory. We sought to test whether training early visual processing would improve visual memory and facial affect recognition by targeting a well-characterized visual deficit in schizophrenia: visual backward masking (VBM). The deficit is so common in schizophrenia, and in non-affected family members to a lesser degree, that it is viewed as an endophenotype. The VBM deficit, however, can normalize as we have previously shown. In our prior open-label pilot study, individuals with schizophrenia (Surti and Wexler, 2012) made substantial gains in VBM performance with rudimentary computerized VBM training. The VBM improvements were also accompanied by improvements in visual memory.

To test the hypothesis that improved early sensory training could lead to other cognitive gains in the same domain, we conducted a randomized control study with a new, more sophisticated computerized VBM training program, and compared it to an active control condition. We expected that visual memory and facial affect recognition would improve with the novel visual training (VT), and that VBM would improve with the VT as well.

**Methods:**

23 individuals with stable schizophrenia or schizoaffective disorder were randomized to receive 20 sessions of VT or an active control. The VT consisted of VBM training with multiple levels of difficulty, adaptive tracking, virtual rewards, and a variety of letters, numbers, and shapes to train different areas of the visual field. The active control condition was a commercially available computerized typing tutorial (TT) with animation, game narrative, and multiple typing activities. Participants were tested before and after training with: the Matrics Cognitive Consensus Battery (MCCB), including the Brief Visuospatial Memory Test-Revised (BVMT) as the study’s primary outcome; the Profile of Nonverbal Sensitivity (mini-PONS) to assess non-verbal social cues; standardized VBM tests; and typing assessments. Repeated measure ANOVAs were conducted in SPSS24 after checking for normality.

**Results:**

22 of 23 individuals completed the study, and by participants’ reports, both interventions were well tolerated, equally enjoyable and equally motivating, though the VT was slightly more frustrating for participants. Even when co-varying for education, which was higher in the VT group, there were no condition by time interactions for the BVMT, the mini-PONS, overall MCCB, or typing ability. There was a significant condition by time interactions for VBM performance (F = 5.8, p =0.028), with a substantial improvement in the VT group (Cohen’s d = 0.54; p=0.004).

**Discussion:**

Patients with schizophrenia equally tolerated a computerized visual training designed in-house and an off-the shelf highly gamified control training, but only the visual training, specifically designed for individuals with schizophrenia, had effects on the trained task. The effects of the visual training did not generalize to visual memory, facial affect recognition, or global cognition, so further work is needed to facilitate generalization.

